# Evaluating the Impact of Pre-Configured Uplink Resources in Narrowband IoT

**DOI:** 10.3390/s24175706

**Published:** 2024-09-02

**Authors:** Muhammad Tahir Abbas, Karl-Johan Grinnemo, Anna Brunstrom, Pascal Jörke, Johan Eklund, Stefan Alfredsson, Mohammad Rajiullah, Christian Wietfeld

**Affiliations:** 1 Department of Mathematics and Computer Science, Karlstad University, 65188 Karlstad, Sweden; karl-johan.grinnemo@kau.se (K.-J.G.); anna.brunstrom@kau.se (A.B.); johan.eklund@kau.se (J.E.); stefan.alfredsson@kau.se (S.A.); mohammad.rajiullah@kau.se (M.R.); 2 Communication Networks Institute (CNI), TU Dortmund University, 44227 Dortmund, Germany; pascal.joerke@tu-dortmund.de (P.J.); christian.wietfeld@tu-dortmund.de (C.W.)

**Keywords:** CIoT, energy efficiency, PURs, EDT, latency

## Abstract

Deploying Cellular Internet of Things (CIoT) devices in urban and remote areas faces significant energy efficiency challenges. This is especially true for Narrowband IoT (NB-IoT) devices, which are expected to function on a single charge for up to 10 years while transmitting small amounts of data daily. The 3rd Generation Partnership Project (3GPP) has introduced energy-saving mechanisms in Releases 13 to 16, including Early Data Transmission (EDT) and Preconfigured Uplink Resources (PURs). These mechanisms extend battery life and reduce latency by enabling data transmission without an active Radio Resource Control (RRC) connection or Random Access Procedure (RAP). This paper examines these mechanisms using the LENA-NB simulator in the ns-3 environment, which is a comprehensive framework for studying various aspects of NB-IoT. The LENA-NB has been extended with PURs, and our analysis shows that PURs significantly enhance battery life and latency efficiency, particularly in high-density environments. Compared to the default RAP method, PURs reduce energy consumption by more than 2.5 times and increases battery life by 1.6 times. Additionally, PURs achieve latency reductions of 2.5–3.5 times. The improvements with PURs are most notable for packets up to 125 bytes. Our findings highlight PURs’ potential to enable more efficient and effective CIoT deployments across various scenarios. This study represents a detailed analysis of latency and energy consumption in a simulated environment, advancing the understanding of PURs’ benefits.

## 1. Introduction

The Cellular Internet of Things (CIoT) is a rapidly evolving field, and the integration of the Narrowband Internet of Things (NB-IoT) has brought a significant transformation to enable efficient and low-power communication across various applications [1]. NB-IoT is widely used [2] due to its ability to handle small data packets while sustaining a battery life of up to 10 years, with a battery capacity of 5 Wh. Due to these features, NB-IoT has become an essential element in the world of CIoT connectivity [3].

Guided by the standards set forth by the 3rd Generation Partnership Project (3GPP) [4], NB-IoT represents the response to the urgent need for a wireless communication solution tailored to the unique requirements of CIoT devices. These requirements, including extended coverage, minimal power consumption, and cost-effectiveness, have driven the development and standardization of these technologies [5]. The commitment of 3GPP to establishing a strong, global framework for NB-IoT underscores its significance in the modern connectivity landscape. Whether in smart cities, agriculture, healthcare, or industrial automation, NB-IoT technology has applications in numerous scenarios. The deployment of NB-IoT devices continues to grow, highlighting the crucial need to optimize energy consumption to maintain the scalability and reliability of these networks [6]. This is particularly important in NB-IoT, where devices often operate in demanding environments with limited power sources.

To address the challenge related to energy consumption, 3GPP, in releases 13 and 14, introduced power saving mode (PSM), extended discontinuous reception (eDRX), and release assistance indicator (RAI). These strategies effectively balance network connectivity and energy consumption [7,8].

In releases 15 and 16, 3GPP introduced two critical mechanisms, Early Data Transmission (EDT) [9] and Preconfigured Uplink Resources (PURs) [10], to reduce energy consumption further. EDT involves timely data transmission during the Random Access Procedure (RAP) to minimize energy usage. PURs aim to streamline uplink (UL) transmissions by pre-allocating resources, reducing latency, and ensuring more efficient use of network resources. These strategies contribute to the continued success and sustainability of NB-IoT.

This paper aims to explore the intricacies of NB-IoT energy efficiency. It provides a comprehensive study of EDT and PURs and their impact on energy consumption and latency, offering an in-depth look at the operational mechanisms of NB-IoT. We understand there has not been a detailed and realistic assessment of these optimizations.

In a study by Hoglund et al. [10], an analytical model was proposed to evaluate the effectiveness of PURs when sending packets and receiving acknowledgments at different layers. However, analytical modeling has limitations and lacks detail, while simulation offers more realistic and detailed scenarios at the cost of higher computational demands.

To address the analytical limitations, we utilized the ns-3 network simulator to extend LENA-NB [11] and create a realistic environment for studying the behavior of EDT and PURs under various scenarios, including energy consumption, latency, impact of radio propagation models, PUR missed/failed slots, coverage levels, etc. The main contributions of this paper are summarized as follows:

- An implementation of PURs inside LENA-NB.
- A study on PURs on energy efficiency, latency, and scalability from a user and network perspective.
- A comparative study on PURs with EDT and the default RAP, including energy-saving mechanisms. Since our study was conducted within a simulator environment, we argue that they are more accurate than the ones obtained by Hoglund et al. [10].

**Figure 1:**
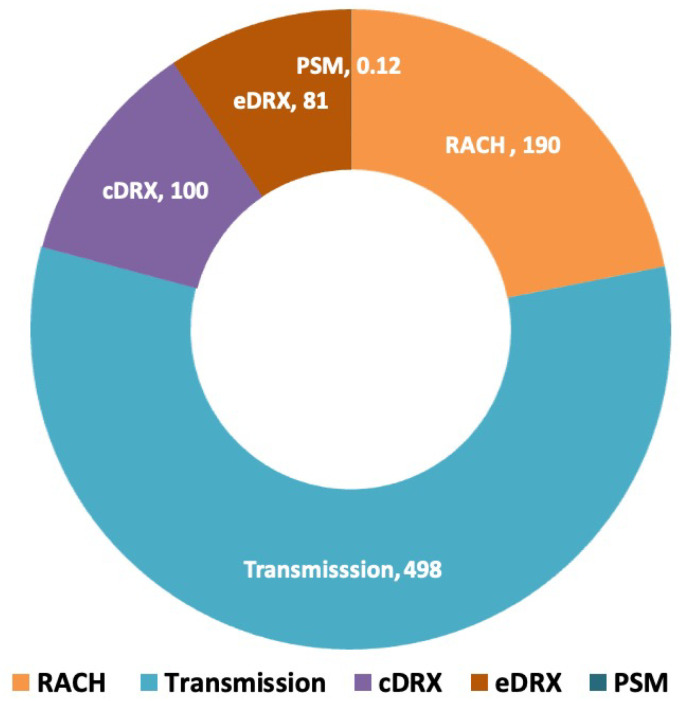
The relative power consumption (mW) among various radio operational states in NB-IoT.

Our study is motivated by the fact that a significant portion of energy consumption in NB-IoT occurs during radio connectivity. This is supported by various studies, including those by Abbas et al. [12], Lukic et al. [13], and Michelinakis et al. [14]. Figure 1 illustrates the energy consumption of an NB-IoT device during its various operational states for a single day, representing a scenario where only one packet is transmitted. It is important to note that a device, throughout its lifespan, circulates through only these operational states. As shown in Figure 1, the initial connection setup, also known as the Random Access Channel (RACH) procedure (orange); packet transmission (light blue); listening to a radio channel (purple); and receiving downlink traffic (red) account for almost all energy consumption. In contrast, the energy spent in PSM mode (dark blue) is negligible and not visible in the figure. Therefore, the opportunity to find energy savings is in the different active radio states and by introducing mechanisms such as EDT and PURs.

The rest of this paper is organized as follows: Section 2 provides a detailed discussion of NB-IoT technology, operating states, and energy-saving mechanisms. Section 3 briefly describes our LENA-NB experimental setup in ns-3. Section 4 provides a detailed analysis of NB-IoT energy-saving mechanisms. Section 5 overviews previously published work on EDT and PURs, while Section 6 concludes the paper.

## 2. NB-IoT

**Figure 2:**
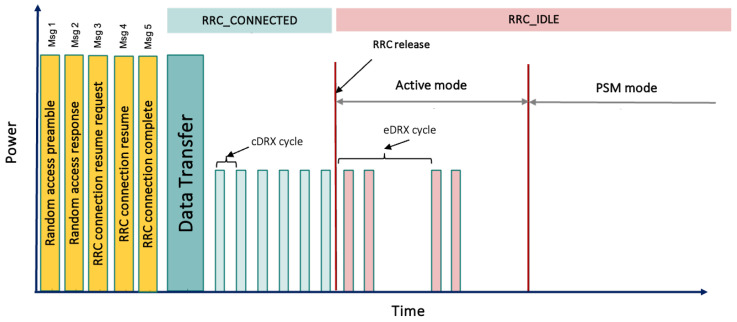
The operational states of NB-IoT, showcasing the stages of the random access procedure and connection setup are highlighted in yellow; the data transmission phase and the CONNECTED state, in green; and the IDLE state, in red.

NB-IoT technology is an evolution that builds upon Long-Term Evolution (LTE) [15,16], a widely adopted standard for high-speed wireless communication, as shown in Figure 2. Collaborative efforts led by 3GPP have resulted in the development of NB-IoT, which specifically addresses the demands of CIoT. NB-IoT operates on narrow bandwidths [17], optimizing spectral efficiency and facilitating seamless integration into existing LTE infrastructure. It uses licensed spectrum frequencies, typically in the sub GHz range, ensuring reliable and secure communication over long distances of 10+ km [18]. This inheritance from LTE provides a solid technological base and allows efficient co-existence with other LTE services.

NB-IoT technology enhances its reach through three distinct Coverage Enhancement (CE) levels—CE Level 0, CE Level 1, and CE Level 2—to improve connectivity in areas with varying signal strengths [19]. These levels, predetermined by the network, dictate the repetition frequency of downlink and uplink traffic to ensure that User Equipment (UE) in low-coverage zones remain connected. CE Level 0 caters to areas with satisfactory coverage, providing a signal boost of +0 dB. For environments requiring more assistance, CE Level 1 steps in with a boost of up to +10 dB. CE Level 2 targets the most challenging conditions by offering a substantial increase of up to +20 dB and permitting up to 128 message repetitions. This advancement increases the maximum coupling loss (MCL) capacity from 144 dB to 164 dB, improving connectivity in “deep indoor” locations where IoT devices may be placed, such as basements and parking structures, areas traditionally problematic for the penetration of 2G, 3G, and 4G (LTE) signals.

NB-IoT technology aims to use energy efficiently, particularly for small data transfers [20]. In release 13, two main methods were introduced to optimize this process [21]. First, the User Plane (UP) optimization, or RRC Suspend/Resume method, simplifies the UE that switch from IDLE to CONNECTED states. It allows devices to “pause” and later “resume” their connection without redoing the entire setup process, saving time and energy. Second, the Control Plane (CP) optimization, or Data-over-NAS method, sends small user data packets through the control channel and bypasses the usual steps of setting up a data connection, further reducing energy use and simplifying data transmission. Both methods were designed to streamline small data transfers; however, CP is more efficient and less energy-intensive [22].

### 2.1. Operational States

In the context of NB-IoT, RRC states play a critical role in managing the communication behavior of UE within the cellular network [8]. NB-IoT RRC states dictate the UE’s operational modes, influencing power consumption, latency, and responsiveness. NB-IoT defines two main RRC states, RRC_CONNECTED and RRC_IDLE, as shown in Figure 2. The RRC_IDLE state represents the state of minimal activity, where the UE conserves power by only periodically listening for paging signals while waiting for potential downlink (DL) data or network commands. On the other hand, the RRC_CONNECTED state signifies an active communication phase, enabling two-way data exchange between the UE and the network. While in RRC_CONNECTED, the UE is more responsive but also consumes more power since it is in what is denoted as the transmission + cDRX state in Figure 1.

The energy efficiency of NB-IoT is strongly based on energy-saving mechanisms embedded within the RRC states, defined by 3GPP release 13. Among these mechanisms, the connected discontinuous reception (cDRX), the extended discontinuous reception (eDRX), and the power saving mode (PSM) are crucial for minimizing energy consumption while maintaining connectivity [8,14], as shown in Figure 2. cDRX and eDRX modes strategically manage the UE’s reception cycles, allowing it to periodically wake up to listen for incoming signals while spending the majority of time in a low-power dormant state. These mechanisms balance the trade-off between responsiveness and power efficiency. However, PSM is designed mainly for applications with infrequent communication needs. It allows the UE to detach from the network and enter an extended sleep mode, significantly conserving energy. The UE re-establishes communication upon awakening, effectively reducing the network signaling overhead. These energy-saving mechanisms are integrated into NB-IoT’s RRC states, contributing to the technology’s ability to support diverse IoT applications with varying energy constraints.

Based on these improvements, subsequent 3GPP releases, such as release 15 [23] and release 16 [24] focused on mitigating signaling overhead and power consumption through innovative solutions such as UE-based EDT and PURs. These solutions include CP and UP CIoT Evolved Packet System (EPS) enhancements. Unlike the conventional five-step default RAP [25], illustrated in Figure 2, the EDT approach introduces a different method. In this approach, the UE signals its intention to transmit small data during Msg 3. On the other hand, PURs further streamline the process by enabling direct transmission in Msg 3, without the need for Msg 1 and Msg 2. These innovations mark significant strides in optimizing the NB-IoT handling of small data exchanges and are discussed in more detail below.

#### 2.1.1. Early Data Transmission

To improve the power efficiency of UE, particularly those that require infrequent small data transmissions in the UL, EDT has been introduced in 3GPP release 15 [11,23]. This approach reduces the power consumption for UE sending sporadic small data packets by enabling data transmission during the RAP. EDT applies to UP and CP solutions, with differences elaborated later in this section.

**Figure 3:**
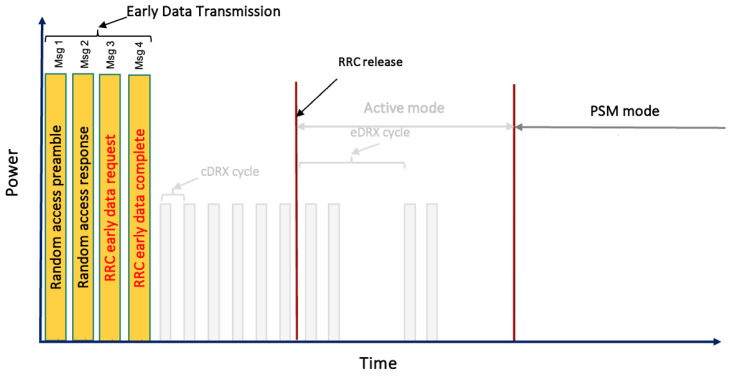
The NB-IoT Uplink Early Data Transmission procedure according to 3GPP release 15, highlighting the transmission of data in Msg 3 and the reception of acknowledgment by the UE in Msg 4.

**Figure 4:**
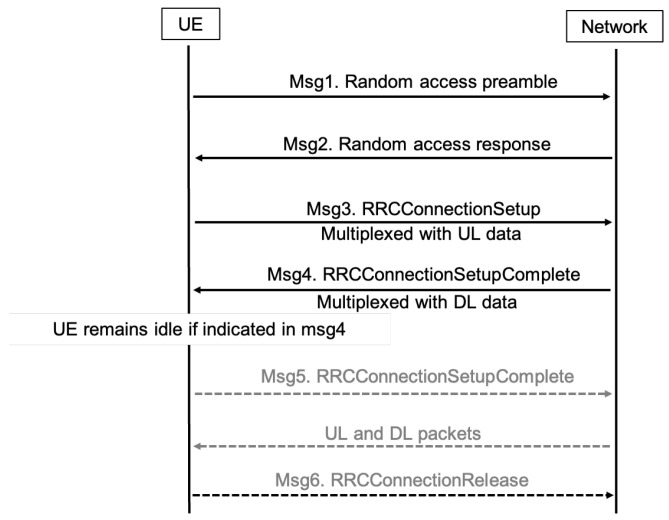
The sequence of messages for establishing connections and transmitting packets, detailing the default RAP from Msg 1 to Msg 6, EDT from Msg 1 to Msg 4, and PURs from Msg 3 to Msg 4.

To implement EDT, UE need to signal their intent to engage in data transmission during Msg 3. Compared to release 13, where the eNodeB lacked prior knowledge of the intentions of the UE data transmission, the EDT mandates using a specific (N)PRACH preamble dedicated to the EDT signaled in Msg 1. Furthermore, the network disseminates the maximum Transport Block Size (TBS) that Msg 3 in EDT can utilize. Over-the-air security poses another consideration. In release 13, data bearer security is established after RAP completion, but for EDT, security must be established during Msg 3 transmission. Mechanisms to achieve this are detailed subsequently. In the RRC_IDLE state, if a UE does not have more data than the broadcast maximum, it may employ EDT, as shown in Figure 3 and Figure 4.

In the UP solution for EDT within release 15, the UE application-specific (AS) context facilitates data resumption and signals radio bearers and security context for user plane data transmission. Compared to the default RAP, where these procedures took place after Msg 4 reception, the UP solution for EDT entails the UE activating suspended data bearers for Msg 3 transmissions, resuming security, and allowing early DL data transmission.

The CP solution for EDT allows UL data within the initial NAS message “CONTROL PLANE SERVICE REQUEST”, transmitted via the RRCConnectionSetupComplete. The objective is to reduce signaling overhead, latency, and UE power consumption. In the CP EDT mechanism, Msg 3 and Msg 4 accommodate NAS messages containing UL and DL user data, ensuring data delivery before Msg 5 while keeping the UE in RRC_IDLE mode.

However, the CP EDT mechanism faces challenges. Unlike the UP EDT approach, it lacks AS security. It employs NAS messages piggybacked on RRC messages through an SRB0 (signaling radio bearer), which can cause issues, especially for Msg 4 with DL data, potentially leading to UE transitioning to RRC_IDLE mode without guaranteeing successful DL data delivery.

In summary, EDT reduces the need to enter the connected state and, therefore, improves the power efficiency of the UE for small, infrequent data transmissions. The UP and CP solutions differ in handling data transmission during the RAP, with considerations for security and UE state transitions.

#### 2.1.2. Preconfigured Uplink Resources

**Figure 5:**
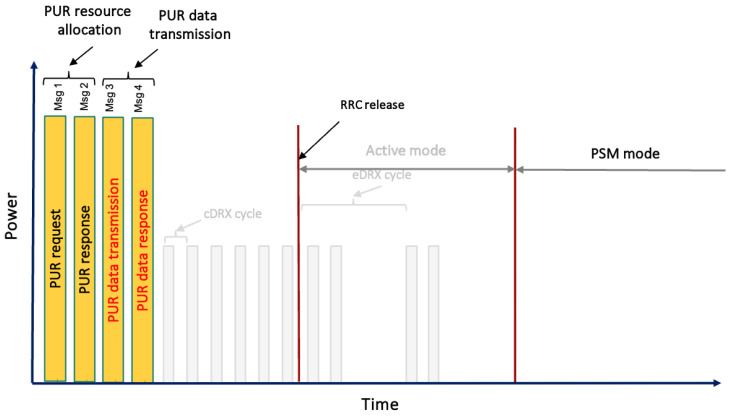
The NB-IoT PUR process as defined in 3GPP release 16, detailing a streamlined two-message protocol where Msg 3 (PUR data transmission) is used for data transmission and Msg 4 (PUR data response) for receiving the acknowledgment.

The objective of employing PURs is to further enhance the efficiency of signaling, a goal distinct from the approach of EDT [24]. The initial phase comprises Msg 1 and Msg 2, which initialize contact with the eNodeB and equip the UE with a valid Timing Advance (TA) for UL synchronization. It is important to note that the initial phase only needs to be completed once, until the UE moves into the proximity of a different eNodeB or the timer for the resources assigned to the UE expires. Subsequently, Msg 3 facilitates the first scheduled UL transmission, housing both an RRC message and a UL data payload. Ultimately, Msg 4 functions to acknowledge UL data transmission, address contention, potentially convey DL data payloads, and orchestrate UE transitions to the CONNECTED state, as shown in Figure 5. It is important to note that PURs achieve their full potential when the User Equipment remains stationary. However, if the UE moves from the proximity of one eNodeB to another, it must acquire new resources each time it needs to transmit a packet. This process is no different from the mechanism used by EDT.

The strategy behind pre-configuring the UE with UL radio resources, under the assumption of a valid TA, is to eliminate the necessity for Msg 1, Msg 2, and conventional connection establishment procedures. The activation of PURs involves dedicated RRC signaling while the UE is in the CONNECTED state, encompassing the acquisition of UE-specific radio resources and a Radio Network Temporary Identifier (PUR C-RNTI), among other parameters.

A comprehensive PUR configuration incorporates periodic time-frequency resources, a Modulation and Coding Scheme (MCS), a Transport Block Size (TBS), PUSCH (Physical Uplink Shared Channel) repetitions, Demodulation Reference Signal (DMRS) configurations, and power control parameters. Triggers for UE configuration with PURs include transmitting a PUR Configuration Request message by the UE to an eNB while in an RRC_CONNECTED state or network-driven activation based on subscription data or identified periodic traffic patterns.

Before executing a PUR transmission, the UE evaluates the validity of the TA through an individual or combined-attribute evaluation, which includes whether the serving cell has changed, the expiration of the PUR TA timer, and variations in received signal power (RSRP). The successful receipt of UL PUR transmissions by the eNB can lead to the conclusion of the PUR procedure in only two messages, UL and DL, recognized through layer-1 signaling in DL Control Information (DCI) or layer-2/3 signaling in an RRC message.

The adaptable periodicity of PURs reflects their suitability for periodic traffic scenarios. However, the configuration flexibility allows the UE to skip transmitting for several consecutive PUR occasions before automatic configuration release.

Unlike EDT, the successful reception of UL PUR transmissions can sometimes be adequately acknowledged through layer-1 signaling if there are no pending DL data. Such acknowledgments might include adjustments to TA, the number of PUSCH repetitions, or even an indication to employ EDT or the ordinary connection establishment procedure. In alternative scenarios, responses to UL PUR transmissions might be conveyed via layer-2/3 signaling (RRC messages) not only for acknowledgments but also for downstream user data transmission, PUR configuration adjustments, and potential UE state transitions.

Two distinct schemes exist for PUR transmissions: dedicated PURs and shared PURs. In the former, UL time-frequency resources are exclusively allocated to a single UE at any given time, particularly suitable for UEs transmitting periodically in the UL. Shared PURs allow the concurrent use of the same UL resources by up to two UEs, distinguished through orthogonal demodulation reference signal sequences. This approach accommodates both periodic and pseudo-varying traffic patterns. However, to prevent excessive interference, shared PURs’ effectiveness are limited to low-Signal-to-Interference-and-Noise Ratio (SINR) regimes.

A cap on the number of UEs employing shared PURs is implemented to manage the potential risk of DL congestion, as simultaneous DL transmissions cannot overlap. The shared PURs additionally require the assignment of distinct PUR C-RNTIs for each of the two UEs transmitting simultaneously. In contrast, dedicated PURs allow te use of a single PUR C-RNTI by multiple UEs, as their use of resources occurs at different points in time. PURs employ a single Hybrid Automatic Repeat reQuest (HARQ) [26] process and can be combined with other features to enhance performance.

## 3. Simulation Environment

The main reason for conducting a simulation study is that while it is clear from the standardization process that EDT and PURs can help reduce energy consumption and improve latency, there is currently no implementation of EDT or PURs by network operators, and no end devices support them. Based on our experience, network operators and end devices currently support release 13 and 14 features. LENA-NB [11] offers a simulation framework with a detailed implementation of radio access technology for NB-IoT, including control plane optimization [3] and EDT. Furthermore, LENA-NB includes several NB-IoT features to enhance network efficiency and reduce device costs. These features include the following:

- A connection resume procedure: An important aspect of LENA-NB is implementing the connection resume procedure, which is part of the RRC connection setup procedure. This functionality lets devices quickly reconnect to the network without redoing the entire setup process. The implementation begins with the device receiving a unique resume ID during the RRC Connection Release phase, crucial for reinitiating a connection without a full setup. A data inactivity timer monitors transmission inactivity, triggering the Connection Release procedure when necessary. This process not only saves the specific entities of the device to the resume ID but also ensures efficient resource management by releasing the C-RNTI and cleaning up pending events. Upon attempting reconnection, the device uses the saved resume ID to restore its connection state, significantly reducing the time and resources required for network re-entry.
- Cross-subframe scheduling In recognizing the absence of an NB-IoT-specific error model, LENA-NB employs a pragmatic approach using a lookup table derived from MATLAB NB-IoT Block Error Rate (BLER) simulations. This table assists in selecting optimal uplink and downlink configurations that meet BLER standards under various channel conditions, optimizing the simulator’s performance across a wide range of scenarios.
- A NB-IoT-specific energy state machine: To provide a detailed simulation of power efficiency, LENA-NB implements the eDRX and PSM techniques at the RRC level. The simulator incorporates an energy state machine that monitors the energy states of a device across different operations, including sending and receiving on various channels and modes, e.g., NPRACH, NPDCCH, NPDSCH, and NPUSCH. This state machine is critical in analyzing and optimizing energy use, drawing on a real-world power consumption data (Quectel BG95 NB-IoT/eMTC module [27]) to provide an accurate and comprehensive energy consumption analysis.

These implementations and modifications are designed to provide a comprehensive understanding and evaluation of UE performance and efficiency in various operational scenarios.

**Figure 6:**
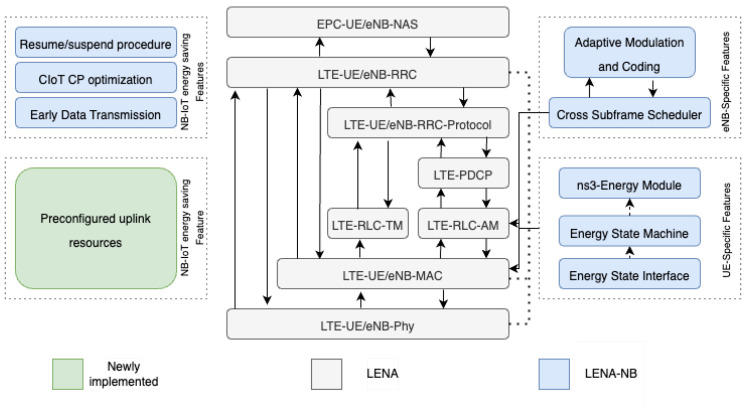
The LENA-NB protocol stack with the resume/suspend extension of Jörke et al. [11], CIoT-OPT, EDT, and our extension of the stack with PUR.

**Figure 7:**
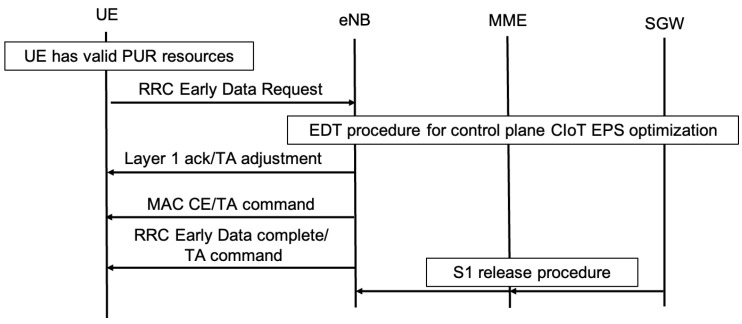
A message sequence diagram depicting the process of packet transmission and acknowledgment during the PUR procedure within the LENA-NB simulation environment.

In our experiments, the simulator was extended with the implementation of PURs [28], as shown in Figure 6. This addition allows a UE to request specific radio resources, such as packet intervals, TBS, and offset. This request is sent by a UE RRC (ueRRC) to eNodeB RRC (enbRRC) before starting transmission, as shown in Figure 7. PURs were designed to handle a maximum packet size of 125 bytes. Packets larger than this limit must be segmented for transmission via PURs; otherwise, the system reverts to the default RAP for managing larger packets. Upon receiving a resource request, the enbRRC passes it to the eNodeB MAC layer (enbMAC), which then forwards it to the scheduler. The scheduler reserves all PUR-specific resources in the UL grid to ensure that these are not allocated to other transmissions. The scheduler then communicates the scheduling information and the assigned resources to ueRRC. It is worth mentioning that resource allocation was predetermined offline before the simulation begins. This pre-allocation is necessary because the eNodeB assigns resources to a UE for a fixed duration, determined by a timer. However, the timing of packet transmissions by the UE, based on the assigned PUR resources, is adjustable. Once a UE has valid PURs, it can transmit data directly to eNodeB.

**Figure 8:**
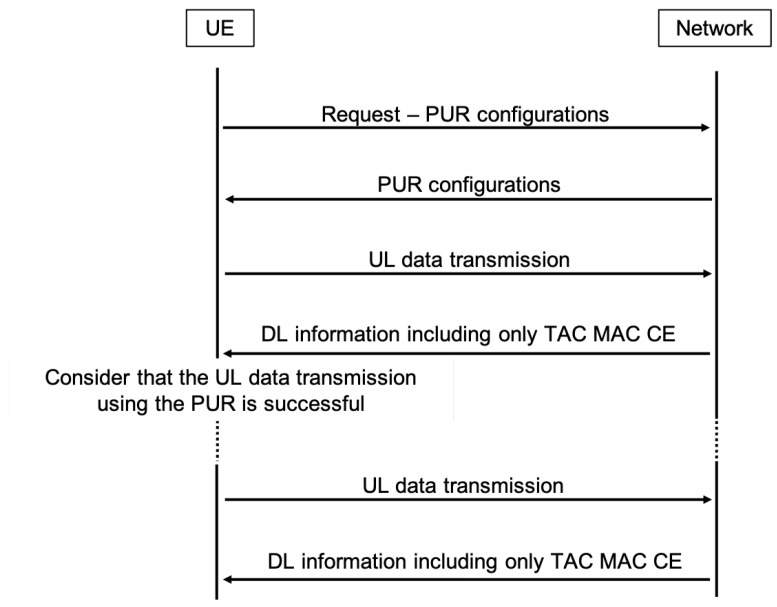
A sequence diagram for a UE-initiated PUR procedure.

In addition, it is worth mentioning that setting up a UE with PURs can be achieved in two ways. First, the UE can initiate the process by sending a PUR request to an eNodeB. Alternatively, the network can trigger the configuration based on factors like subscription details or the recognition of a regular traffic pattern. In our simulations, we only considered the PURs triggered by a UE, as shown in Figure 8.

## 4. Experiments

In evaluating PUR’s functionality, we conducted a series of experiments focusing on different parameters. Table 1 outlines the parameters affecting the behavior of the UE at various CE levels, while Table 2 lists the parameters relevant to our simulation setup. The UEs were divided equally among three coverage conditions: outdoors, indoors, and “deep indoor” environments, such as basements.

In the default RAP, a UE sends one message per day and enters an IDLE state after a 20 s wait in connected mode. For EDT and PURs, a UE moves into the IDLE state immediately after sending an uplink packet and receiving the corresponding acknowledgment. The radio signal is weaker for “indoor” and “deep indoor” placements, requiring more repetitions for the same message, as shown in Table 1, columns CE1 and CE2. The path loss of the UE is calculated using the Winner+ channel model, which simulates both line-of-sight (LOS) and non-LOS propagation conditions. Additionally, the configurations include building losses of 15.4 dB for “indoor” and 20.9 dB for “deep indoor”, as derived from [29].

Our simulations lasted 45 min, but we concentrated our evaluation on a central 15 min interval. The initial 15 min period usually produces little results because UE are scheduled in an empty cell, resulting in excellent transmission conditions. However, after this period, the ongoing transmissions from the earlier UE create a more realistic environment and yield significant results. To account for transmissions initiated but not completed within this time frame, we simulated an additional 15 min, introducing new transmissions to maintain channel activity and allowing earlier UE to complete their transmissions. However, this does not apply to PURs since eNB allocates resources beforehand, reducing the dependence on interference from other UE.

**Figure 9:**
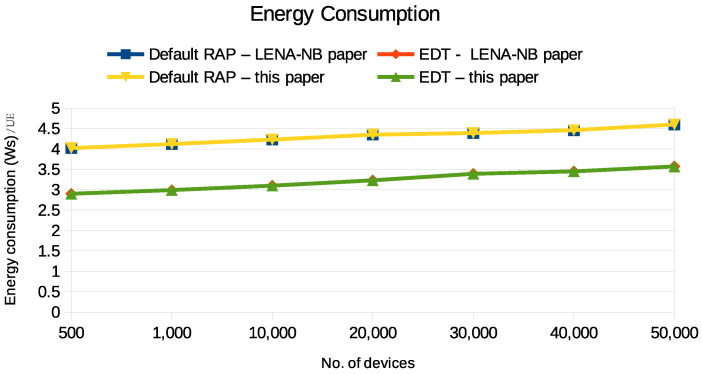
The energy consumption of EDT and the default RAP are shown, including the measured outcomes from this study, as well as those referenced from the LENA-NB paper [11].

Initially, we validated the LENA-NB simulator extended with PURs against the original LENA-NB simulator [11]. The results from this validation are depicted in Figure 9. We conducted a comprehensive comparison, which included generating results for 50,000 UEs to evaluate energy consumption. The results indicate that the energy consumption levels of EDT and the default RAP in our experiment were very similar to those found in [11]. After validating our simulation setup, our research primarily delves into PUR—an area that we consider crucial for understanding energy efficiency in the NB-IoT. Therefore, in the following sections, we will delve deeper into the analysis of PUR, emphasizing its significance and potential impacts on network performance.

### 4.1. Scalability

The introduction of PURs significantly improves the network scalability in NB-IoT by allowing more devices to connect compared to the default RAP and EDT. This mechanism is particularly effective for managing uplink communication from multiple IoT devices. By pre-allocating uplink resources, PURs enable UE to transmit data without dynamic resource scheduling, which can be complex and resource-intensive, especially in large-scale deployments. In addition, the simulations showcase eNB’s capability to serve up to 62,000 UEs under idealized radio conditions in an idle environment within one day. Additionally, a dedicated PUR setup with a 10% PUSCH BLER can manage up to 52,000 UEs.

The DL spectrum usage in the default RAP is significantly impacted by system broadcasts, even in the absence of user data. Specifically, broadcasts such as the Master Information Block (MIB), Narrowband Primary Synchronization Channel (NPSS), Narrowband Secondary Synchronization Channel (NSSS), SIB1-NB, and additional System Information (SI) messages consume 30% of the DL spectrum. This leaves only 70% available for user-specific transmissions. As the number of connected devices increases, the spectrum usage ratio inevitably rises. However, the introduction of EDT and PURs offers a substantial reduction in the overhead associated with small data transmissions. This reduction in spectrum usage, by a factor of 3.7 [11] and 5.6, respectively, effectively increases cell capacity by the same factor, making PURs a highly recommended approach.

In addition to DL constraints, the UL spectrum also plays a crucial role in determining cell capacity. Although the UL does not carry broadcast information, its available spectrum is reduced by the configuration of RA windows, which reserve portions of the spectrum regardless of actual RA activity. Therefore, a careful balance must be maintained between allocating sufficient intervals for RA windows and preserving spectrum for application data transmission. In well-configured scenarios, RA collisions are minimal, ensuring that the successful data transfer remains at optimal levels. However, as the number of connected devices increases further, the likelihood of RA collisions rises, potentially preventing devices from accessing the cell. In such cases, it will be necessary to optimize the RRC configuration to maintain efficient cell access and performance.

### 4.2. Energy Consumption and Latency

**Figure 10:**
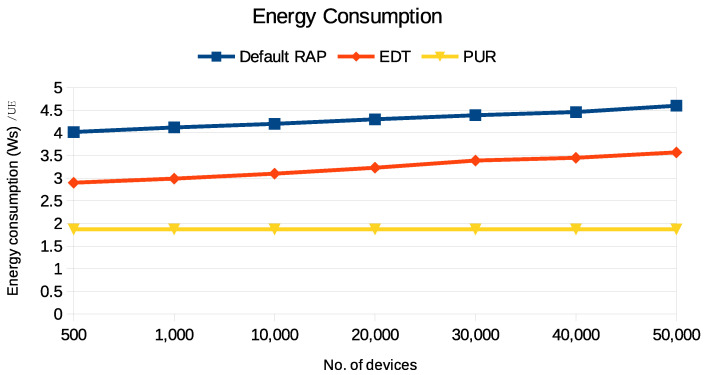
A comparison of average energy consumption/UE for default RAP and different NB-IoT optimization techniques.

The graphs in Figure 10 show the energy consumption of the default RAP, EDT, and PURs. Both the default RAP and EDT show a linear increase in energy usage as the number of UE in the network increases. However, EDT is more energy efficient, consuming approximately 3.5 Ws per UE for 500 UEs and slightly above 4 Ws per UE for 50,000 UEs. PUR optimization exhibits the lowest energy consumption at 2.5 Ws per UE. It optimizes the scheduling of uplink transmissions and enhances control channel resource allocation. The linear energy consumption observed in the default RAP and EDT is attributed to the random intervals at which packets are transmitted, causing devices to wait for ongoing transmissions to complete. In contrast, EDT’s constant energy consumption line is due to the pre-assigned time and frequency resources, eliminating the need for competition. Moreover, the linear energy consumption observed in the RAP and EDT, causing devices to wait for ongoing transmissions to complete, is due to the random time intervals at which packets are transmitted. In contrast, the constant energy consumption line for PURs is due to the pre-assigned time and frequency resources, eliminating the need for competition.

**Figure 11:**
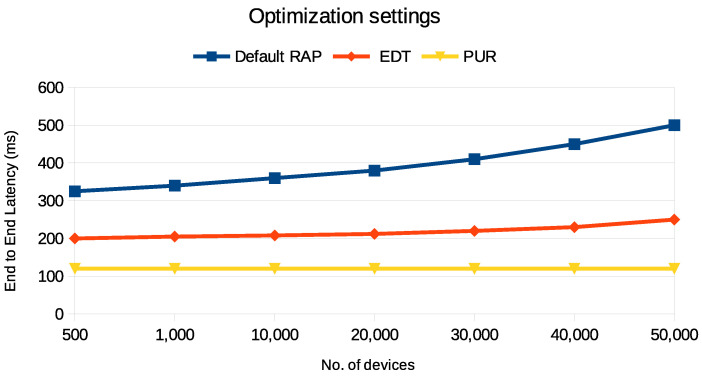
A comparison of end-to-end latency for three different NB-IoT optimization techniques.

Figure 11 presents the end-to-end latency performance in NB-IoT networks under the two optimization settings, EDT and PURs. EDT and PURs demonstrate improved latency over the default RAP, particularly when more UE are served, but their mechanisms differ significantly. EDT involves multiple communication steps between the UE and the network, completing the transmission of the packet in four messages. On the other hand, PURs only use two messages to complete communication, assigning radio resources to UE beforehand, reducing the time and complexity involved in the communication process. Thus, PURs minimize latency and reduce the rate at which latency increases with the addition of more UE, making it a crucial optimization technique for large-scale IoT deployments.

**Figure 12:**
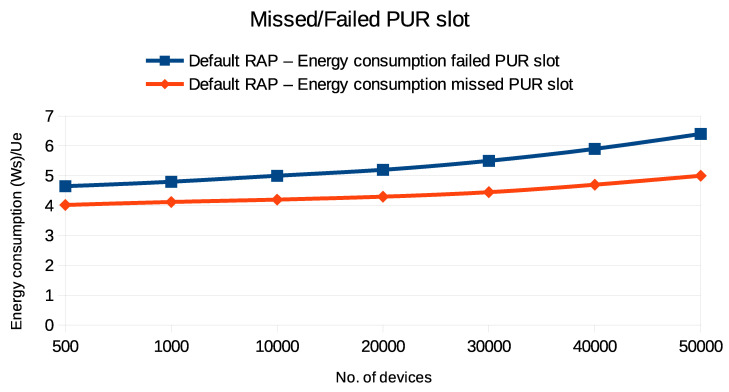
Energy consumption during PUR fallback to default RAP when PURs miss or fail to send data during PUR slot.

**Figure 13:**
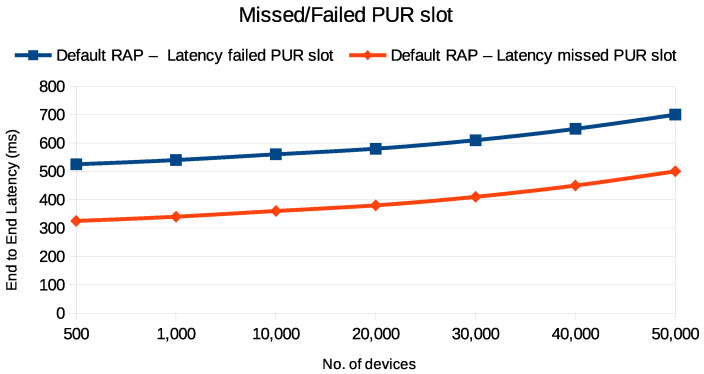
End-to-end latency during PUR fallback to default RAP when PURs miss or fail to send data during PUR slot.

The graphs in Figure 12 and Figure 13 demonstrate the impact of missed or failed PUR slot transmissions on energy consumption and end-to-end latency in UE. Figure 12 illustrates two scenarios: one where the UE reverts to the default RAP after a failed PUR attempt and another where the UE misses the PUR slot and falls back to the default RAP. It is evident that the failed PUR attempt and the fallback to the default RAP result in significantly higher energy consumption compared to the missed PUR slot. Similarly, Figure 13 reveals that the latency is greater when the UE switches to the default RAP following a failed PUR attempt, compared to the scenario in which the PURs miss the slot. Both figures indicate that energy consumption and end-to-end latency increase as the number of devices increases, highlighting the inefficiency of a failed PUR transmission.

This increased energy consumption and latency is due to the additional energy used during the failed PUR attempt and the subsequent less efficient default RAP transmission, leading to delays in resource acquisition and overall system inefficiency. It is important to note that these scenarios also assume the presence of adequate synchronization (i.e., a valid TA) for PURs to be effective. However, in practical deployments, especially in devices with low-accuracy synchronization sources, timing drift can lead to missed PUR slots or failed attempts, further exacerbating energy consumption and latency. Additionally, variations in the channel conditions can introduce further challenges, potentially reducing the reliability of PURs and necessitating more frequent fallbacks to the RAP. These factors must be considered when evaluating the effectiveness of PUR schemes, particularly in networks with varying loads and device types.

### 4.3. Propagation Models

**Figure 14:**
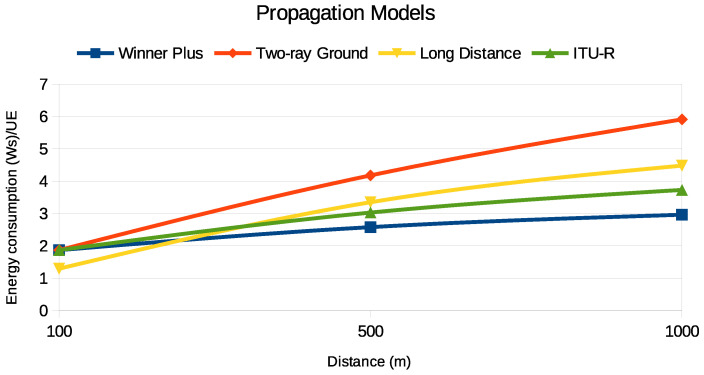
Energy consumption of PURs under different propagation models.

**Figure 15:**
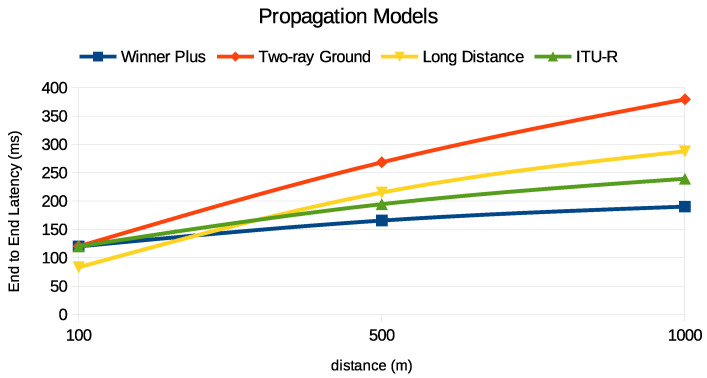
End-to-end latency of PURs under various propagation models.

We examined how various propagation models affect the performance of PURs, focusing particularly on energy consumption and end-to-end latency, as illustrated in Figure 14 and Figure 15. We considered four models: Winner Plus [30], Two-ray Ground [31], Long-Distance Pathloss [32], and ITU-R [33]. Each model has distinct characteristics influenced by environmental factors and propagation effects.

The Winner Plus model, commonly used in urban macro-cell environments, shows a moderate increase in energy consumption and latency with distance due to realistic urban propagation effects like shadow fading and multipath reflections. On the other hand, the Two-ray Ground model, which considers both direct and ground-reflected paths, results in significantly higher energy consumption and latency over long distances due to destructive interference and signal loss.

The Long-Distance Pathloss model demonstrates a logarithmic increase in energy consumption and latency, reflecting compounded signal losses and increased power requirements over extended distances. Meanwhile, the ITU-R model, with its power-law path loss relationship, provides a balanced approach with moderate energy consumption and latency increases, making it suitable for various environments.

While these findings highlight the influence of the chosen propagation model on energy consumption and latency, it is important to note that in practical scenarios, the energy consumption of the UE is also significantly affected by its operational behavior in response to the network conditions. For instance, the number of repetitions required to achieve reliable communication, adjustments in CE levels, and other adaptive behaviors of the UE play a crucial role in determining the actual energy consumption. Therefore, the selection of an appropriate propagation model should be complemented by a consideration of these UE behaviors to optimize the trade-off between energy efficiency and latency in NB-IoT networks.

### 4.4. Packet Size

**Figure 16:**
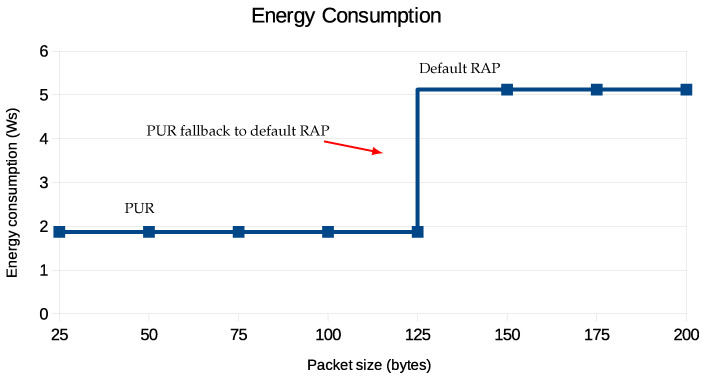
PUR fallback to default RAP when transport block size exceeds assigned resources.

**Figure 17:**
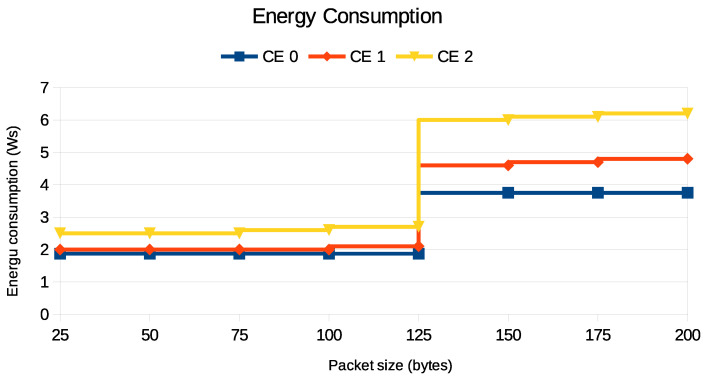
PUR behavior under different radio coverage with varying packet size.

NB-IoT is a crucial technology in IoT applications due to its low power consumption. However, the energy efficiency of this technology is significantly influenced by the size of the packet, as shown in Figure 16 and Figure 17.

Figure 16 illustrates the energy consumption for two different methods of packet transmission: PURs and the default RAP. The PUR method efficiently handles packet sizes up to 125 bytes, maintaining relatively stable energy consumption. However, once the packet size exceeds 125 bytes, the system switches to the default RAP, resulting in a sharp increase in energy consumption. For packet sizes between 125 and 200 bytes, the energy consumption remains high but stable, indicating the less energy-efficient mode of the default RAP.

Figure 17 shows the trade-off between different coverage levels and energy consumption for UEs that use PURs. When UE need to cover a wider area, they use more energy, especially when the packet size is larger. This becomes especially noticeable when a UE has to use PURs to send packets larger than 125 bytes, causing the packets to be split and sent in multiple PUR occasions as PURs only support a maximum packet size of 125 bytes.

When packets exceed 125 bytes, an additional PUR occasion is necessary. This requires another round of transmission and acknowledgment, effectively doubling the energy consumption compared to when the packet size remains at or below the threshold. Despite this, using PURs for packet transmission is still more energy-efficient than the default RAP, especially for CE Levels 0 and 1, as shown in Figure 16. However, it is important to note that for CE Level 2, PURs’ energy consumption increases. The reason is that a UE with poor coverage ends up sending more repetitions of the same packet in the lower layers, as shown in Table 1, to increase the probability of the successful transmission of the packet. This has implications for the deployment of NB-IoT solutions, as UE operating in areas that require CE Level 2 will have shorter battery life and may need more frequent battery replacement. This increased energy consumption can be attributed to the higher power requirements for signal transmission and reception and a higher number of repetitions for the same packet at lower layers.

### 4.5. Expected Battery Life

**Figure 18:**
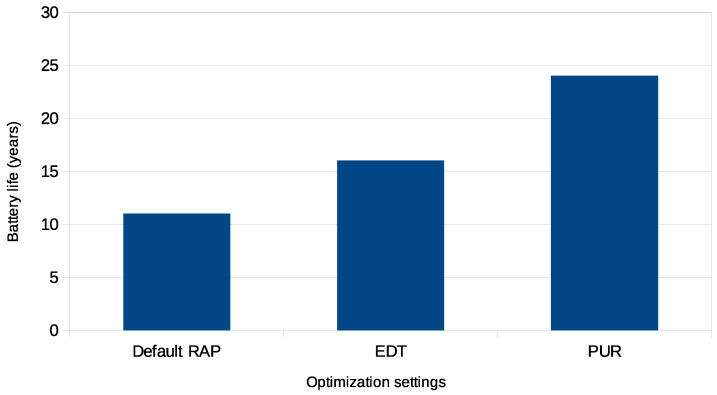
Estimated battery life of an NB-IoT device configured with different optimization techniques.

Figure 18 compares the battery life expectancy of an NB-IoT device under different optimization settings, based on the 5 Wh battery capacity. These simulations involved transmitting 49 bytes of data every 24 h. After transmission, the UE enters the IDLE state, except in the default RAP scenario, where the UE remains awake until the inactivity timer expires before transitioning to the IDLE state.

The figure illustrates that the default RAP has the shortest battery lifespan due to its conventional method, which involves multiple steps for establishing a network connection. This approach requires frequent UE wake-ups and consumes significant energy for communication. On the other hand, EDT improves battery life by 1.6 times compared to the default RAP [11]. EDT achieves this by allowing the UE to send data immediately after the initial access attempt, reducing the need for multiple network interactions. The most significant improvement is observed with PURs, where network resources are allocated in advance. This reduces the need for repetitive access procedures and minimizes the active communication time of the UE, resulting in a 2.1-fold increase in battery life compared to the default RAP.

These optimizations enable extended battery life, which is crucial for the sustainability of UEs in long-term deployments where frequent battery replacements are not practical. The battery life improvements presented in the figure are all related to the 5 Wh battery capacity, emphasizing the importance of efficient network operation strategies to maximize the operational lifespan of IoT devices.

## 5. Related Work

Extensive research has been conducted on optimizing the overall energy consumption of NB-IoT devices. Adjustable parameters such as cDRX, eDRX, PSM [8,12], CP, and UP optimizations [34,35] and the default RAP [13,14] have been explored. Moreover, there are some previous studies on PURs [10,36]; however, these studies mainly rely on theoretical models and analyses. Therefore, additional research is needed to improve our understanding of how PURs impact NB-IoT energy efficiency.

The authors Jorke et al. [11] implemented NB-IoT in the LENA-NB ns-3 framework. In their evaluation of NB-IoT, they showed that EDT could offer up to 2.9 times less latency, 1.6 times longer battery life, and 3.7 times less spectral usage than the standard optimization of the NB-IoT control plane. We heavily rely on their simulation environment as a vital part of our research infrastructure.

Wang et al. [37] showed how EDT and PURs could reduce NB-IoT signaling overhead in industrial IoT systems. Hoglund et al. [10] showed that PURs are the most effective way to reduce UE power consumption when frequent data transmissions with small payloads are involved. For instance, with a 30 s periodicity, a 50 byte payload, and a 43 min eDRX cycle, PURs yield gains ranging from 7% in deep coverage to 50% in good coverage compared to EDT. The authors conclude that PURs significantly reduce signaling exchanges for small-size data transmissions, especially when uplink data transmissions occur hourly or less frequently, and the payload is minimal. Hoglund et al. also compare two PUR transmission schemes, dedicated and shared PURs. Their research shows that shared PURs provide a 100% increase in the utilization of PUSCH resources in low-SINR regimes, while dedicated PURs are more versatile across SINR regimes, making it the preferred choice in several other scenarios.

Prasad et al. [36] introduced SmartPUR, a version of PURs that utilizes Machine Learning (ML) techniques to verify and predict TA for mobile CIoT devices. Their evaluation of SmartPUR demonstrates its capability to significantly minimize fallback rates and reduce power consumption for mobile CIoT devices.

Finally, the paper by Khlass et al. [38] examines two methods for transmitting small amounts of data while the device is inactive. The first method uses RACH, and the second uses Configure Grant (CG). The study compared the two methods and examined their impact on packet delay, power consumption, and signaling overhead metrics. The results showed that the CG-based method is better, with improved power efficiency and reduced latency.

## 6. Conclusions

This paper examined the impact of PURs on NB-IoT energy consumption and compared it with those of EDT and the default RAP mechanism. We tested PURs using the LENA-NB framework in ns-3, and our tests indicated that PUR significantly reduces energy consumption and latency compared to the default RAP and EDT. By pre-allocating network resources, PURs reduced latency by 2.5–3.5 times and energy consumption by 2.1 times compared to the default RAP, while EDT reduced latency by 1.5–2 times and energy consumption by 1.6 times.

Our research shows that using PURs and EDT can significantly improve the energy efficiency and overall performance of NB-IoT networks. Based on our simulation results, we strongly recommend that these optimization techniques be widely adopted in future IoT deployments to create more sustainable and efficient CIoT networks. However, we still need to verify our findings in real-world deployments, which we plan to perform once EDT and PURs are supported in commercial cellular networks. Additionally, we aim to explore the limitations and possibilities of using PURs for large-scale deployments. It will be crucial to assess the scalability of shared and dedicated PURs, and we plan to address this in our future work.

## Figures and Tables

**Table 1:** The configuration of the LENA-NB simulator.

Parameter	CE0	CE1	CE2
rsrp-Thresholds	–	−116 dBm	−128 dBm
nprach-StartTime-r13	256 ms	256 ms	256 ms
nprach-SubcarrierOffset-r13	36	24	12
nprach-NumSubcarriers-r13	12	12	12
nprach-SubcarrierMSG3-RangeStart-r13	twoThird	twoThird	twoThird
maxNumPreamble-AttemptCE-r13	10	10	10
numRepetitionsPer-PreambleAttempt-r13	1	8	32
npdcch-NumRepetitions-RA-r13	8	64	512
npdcch-Offset-RA-r13	zero	zero	zero

**Table 2:** The LENA-NB experiment setup.

Parameters	Settings
Area	4.91 square kilometers
UE deployments	Outdoor, indoor, deep indoor
Attenuation model	Winner plus propagation (10%)
Packet size	49 bytes
Base station height	50 m
eNB and UE antenna	Isotropic Antenna Model
NB-IoT battery capacity	5 Watt-hours/18,000 Watt-seconds
Simulation time	45 min

## Data Availability

The original contributions presented in the study are included in the article, further inquiries can be directed to the corresponding author.
